# Implementation of a national waterborne disease outbreak surveillance system: overview and preliminary results, France, 2010 to 2019

**DOI:** 10.2807/1560-7917.ES.2021.26.34.2001466

**Published:** 2021-08-26

**Authors:** Jerome Pouey, Catherine Galey, Julie Chesneau, Gabrielle Jones, Nathalie Franques, Pascal Beaudeau, groupe des référents régionaux EpiGEH, Damien Mouly

**Affiliations:** 1 Santé publique France, Saint-Maurice, France; 2 Direction générale de la Santé, Ministère des solidarités et de la santé, Paris, France

**Keywords:** waterborne disease outbreaks, health insurance data, medicalised acute gastro-enteritis, spatio-temporal clusters, surveillance system, EpiGEH

## Abstract

**Background:**

Waterborne disease outbreaks (WBDO) associated with tap water consumption are probably underestimated in France.

**Aim:**

In order to improve their detection, Santé publique France launched a surveillance system in 2019, based on the periodical analysis of health insurance data for medicalised acute gastroenteritis (mAGE).

**Methods:**

Spatio-temporal cluster detection methods were applied to mAGE cases to prioritise clusters for further investigation. These investigations determined the plausibility that infection is of waterborne origin and the strength of association.

**Results:**

Between January 2010 and December 2019, 3,323 priority clusters were detected (53,878 excess mAGE cases). They involved 3,717 drinking water supply zones (WSZ), 15.4% of all French WSZ. One third of these WSZ (33.4%; n = 1,242 WSZ) were linked to repeated clusters. Moreover, our system detected 79% of WBDO voluntarily notified to health authorities.

**Conclusion:**

Environmental investigations of detected clusters are necessary to determine the plausibility that infection is of waterborne origin. Consequently, they contribute to identifying which WSZ are linked to clusters and for which specific actions are needed to avoid future outbreaks. The surveillance system incorporates three priority elements: linking environmental investigations with water safety plan management, promoting the systematic use of rainfall data to assess waterborne origin, and focusing on repeat clusters. In the absence of an alternative clear hypothesis, the occurrence of a mAGE cluster in a territory completely matching a distribution zone indicates a high plausibility of water origin.

## Introduction

Waterborne disease outbreaks (WBDO) are still a public health issue worldwide [1-3]. They are generally caused by the microbiological contamination of tap water, and acute gastroenteritis (AGE) is the most common syndrome in affected people. Faced with this issue, many countries have implemented dedicated surveillance systems [1,3-5]. However, notification processes (voluntary or mandatory) vary, as do definitions for WBDO. Standardised information is collected including epidemiological, clinical and, occasionally, biological data, as well as data on the drinking water supply zone (WSZ) in question and operating and distribution incidents. A WSZ refers to a geographically defined area within which water intended for human consumption comes from one or more sources, and where water quality may be considered as approximately uniform. Although most surveillance systems are affected by underdetection, assessments all tend to highlight the same risk factors: rainy events leading to pollution and flooding of the water resource, microbiological vulnerability of the resource, operating incidents (disinfection failure, filtration incident) or a distribution incident (pipeline break, backflow of waste water to the drinking water supply) [6]. Moreover, contributing environmental factors may be aggravated by climate change, thereby increasing the health burden attributable to tap water [7,8].

In France, health authorities notify WBDO to Santé publique France (SpFrance, the French Public Health Agency). SpFrance then investigates the reported issue [9-12]. There is no standard declaration procedure for reporting WBDO. They are usually notified to health authorities through voluntary reporting by general practitioners or pharmacists following official drinking water monitoring results, or following consumer complaints (smell, taste, etc). Rarely, WBDO are also notified through the Food-borne Infectious Outbreak (FIO) mandatory surveillance system, which is also managed by SpFrance. The lack of a specific WBDO surveillance system leads to underestimation of their health impact. Studies based on improving sensitivity, by using health insurance data to record medicalised acute gastroenteritis (mAGE) cases, have proven both their utility in the study of infectious risk attributable to tap water, and their applicability in retrospective WBDO detection systems [13-16].

In this context, SpFrance, in partnership with the Ministry of Health and regional health agencies (ARS), designed a national French WBDO surveillance system based on health insurance data. The 3-year start-up period to test the system commenced in April 2019. Its main objectives are (i) to facilitate the identification and management of WSZ that need to be secured and made safe to protect consumers’ health and (ii) to improve contamination prevention through increased knowledge of WBDO in France and associated risk factors. Furthermore, this new system will provide epidemiological indicators to better estimate the health impact of WBDO.

This article presents the structure and organisation of this new French WBDO surveillance system. We focus on the web-based application EpiGEH, which was specially developed for the system by SpFrance.

## Methods

### Overview of the surveillance system

**Figure 1:**
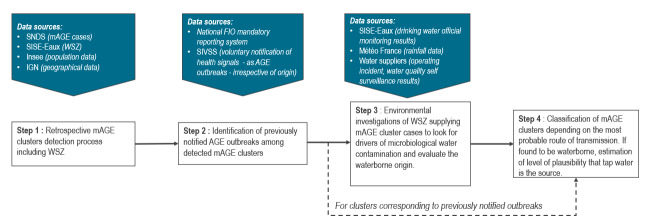
Main steps and data sources of the national surveillance system for waterborne disease outbreaks in France

AGE: acute gastro-intestinal enteritis; FIO: food-borne infectious disease; IGN: French acronym for National Institute for Geographical and Forest Information; Insee: French acronym for National Institute of Statistics and Economic Studies; mAGE: medicalised acute gastro-intestinal enteritis; SISE-Eaux: French acronym for Health and Environment Water Information system; SIVSS: French acronym for Information System for Health Security; SNDS: French acronym for National Health Data System; WSZ: water supply zone.

The main steps of the WBDO surveillance system are:

- Step 1: the retrospective detection of mAGE clusters using a spatio-temporal method. This involves investigating the WSZ involved and selecting clusters for further investigation depending on epidemiological and statistical criteria;
- Step 2: an initial investigation of selected clusters (see Step 1) to identify those which correspond to previously notified (e.g. by health practitioners) AGE outbreaks, and which have already been investigated in the past;
- Step 3: an environmental investigation of clusters not previously investigated (see Step 2) to evaluate whether the contamination is waterborne;
- Step 4: the classification of these (see Step 3) clusters according to most probable transmission route. For clusters suspected to be waterborne, the level of plausibility that tap water is the origin is assessed (Figure 1).

### Zone and population concerned

The new WBDO surveillance system covers all French metropolitan counties and overseas territories (Guadeloupe, Martinique, Guyana, Reunion and Mayotte). All residents older than 1 year are covered. Children younger than 1 year are excluded because consumption of tap water in this age group is marginal in France (breastfeeding and bottled water generally being more common).

### Data sources

#### Health data sources

The National Health Data System (SNDS) database is managed by the French National Health Insurance system. It covers almost all the French population (99%) and records individual sociodemographic information about healthcare services received and pharmacy-based reimbursements for medicines. Partial or full systematic reimbursement for purchased medicines is a key feature of the country’s insurance system [17]. For the WBDO surveillance system, the SNDS is used to identify mAGE cases, defined as follows: any AGE case who consulted a general practitioner and purchased medications prescribed to treat AGE at a pharmacy within 2 days of the consultation. A specific algorithm built in 2011 and regularly updated is used to identify mAGE cases [18,19]. Cases are aggregated according to municipality of residence, date of consultation and age group (1–15 years and > 15 years). A 2-month data consolidation delay is required until mAGE indicators are available, as it takes this amount of time for the health insurance system to update its records.

The Information System for Health Security (SIVSS) is managed by the Ministry of health. This national database centralises, in real time, all health and environmental events voluntarily notified. Notified AGE outbreaks typically occur in nursing homes or schools. Suspected WBDO, voluntarily notified by general practitioners, and tap water restrictions following microbiological tap water contamination, are also recorded. For each notification, investigation results (epidemiological, environmental), if any, are recorded in the SIVSS database.

The National Food-borne Infectious Disease (FIO) Mandatory Reporting System (NFIOMRS) is managed by SpFrance. Health professionals must report FIO to ARS [20]. The FIO notifications include information on cases, symptoms, date of symptom onset and suspected meals.

The SIVSS and NFIOMRS databases are not specific to WBDO and have very low sensitivity for detecting them. They are cross-referenced with detected mAGE clusters to determine whether certain clusters have previously been notified.

#### Environmental data sources

The Health and Environment Water Information system (SISE-Eaux d’alimentation) is managed by the Ministry of Health and regularly updated by ARS. This database includes information on WSZ infrastructure, such as size of each population served at the geographical intersection between WSZ and the municipality. In April 2020, ca 24,200 WSZ supplying 35,573 municipalities were listed throughout all French territory. The SISE-Eaux also centralises all the results, including microbiological analyses, from the official monitoring of raw, treated and tap water. The geographical intersection between WSZ and municipalities is used at the mAGE cluster identification step (Step 1 above), while official data from tap water monitoring are used for environmental investigations of detected clusters (Step 3 above).

Météo-France collects daily rainfall data in all French counties (metropolitan and overseas). These data are used for environmental investigations to identify meteorological events that may be associated with detected mAGE clusters arising from vulnerability of a water resource (particularly for surface water resources or karstic ground waters) and/or lack of adequate tap water treatment. Data on daily precipitation at the nearest weather station to the water abstraction point are required for the current month and 2 months preceding the event in order to perform environmental investigations.

Water suppliers collect information not necessarily included in the SISE-Eaux database, through monitoring of their installations (including malfunctions). They also collect data on tap water quality (self-monitored by the supplier) and complaints from consumers. All these data are voluntarily provided for cluster environmental investigations.

#### Other data sources

The National Institute of Statistics and Economic Studies (Insee) updates the repository of French municipality and census data annually. The National Institute of Geographical and Forest Information (IGN) manages geographical administrative information. The Insee and IGN databases are used to cross-reference the SISE-Eaux and SNDS databases during the mAGE clustering process (Step 1 above).

### Cluster detection

Retrospective mAGE cluster detection is carried out at the county level using the Kulldorff spatio-temporal method [21]. The detection process is optimised using a specific algorithm that considers that all mAGE cases sharing the same WSZ within a period of 4 weeks (i.e. having the same water quality) form a cluster [22]. Given the different possible WSZ–municipality configurations (one WSZ serving only one municipality; more than one WSZ serving only one municipality; one WSZ serving more than one municipalities; more than one WSZ serving more than one municipalities), each detected cluster may be associated with one or more WSZ and/or municipalities. In a simulation study, the positive predictive value and sensitivity of the detection process were estimated at 90% and 73%, respectively, for 2,000 simulated WBDO [23].

Possible confounders such as holiday periods, day of the week, winter season (linked to winter AGE outbreaks) and population density at the municipality level are introduced into the detection process as co-variables of mAGE incidence. A minimum cluster duration of 3 days is also required.

Each detected cluster with a p value < 0.05 is associated with a unique identification number, a list of related WSZ and epidemiological information corresponding to the cluster period: number of observed, expected and excess cases, risk ratio (ratio of observed to expected cases), age distribution between youths (i.e. < 16 years-old) and adults, date of onset and cluster duration.

### Cluster investigations and classification

#### Identification of previously notified AGE outbreaks

Initial investigations of AGE clusters aim to identify those previously notified in the SIVSS database and/or NFIOMRS. When the route of transmission (i.e. food or person-to-person contamination) has already been documented for such clusters, the investigation is stopped, and the cluster is classified according to the documented source.

#### Environmental investigations

Environmental investigations aim to determine the plausibility that contamination is of waterborne origin, and to attribute the level of plausibility. Several criteria are defined to evaluate plausibility [16,24]:

- Malfunctions or malfunction-related events in the WSZ (e.g. microbiological contaminations, chlorine breakdown, etc.) identified during the suspected exposure period (i.e. between 30 and 15 days before the cluster and until the date of the end of the cluster);
- Documented vulnerabilities of the WSZ to microbial risk, based on historical faecal water contamination results at different points (resource, production, distribution);
- External event potentially leading to contamination (such as heavy rain, backflow of contaminated water into the network, etc.) during the suspected exposure period.

The ARS lead the environmental investigations using an investigation form specifically designed for mAGE (Supplementary Table S1) according to criteria set down by the French Ministry of Health. They can also contact water suppliers to obtain additional information needed to complete the form [24]. The completed form is transmitted to the national coordination office via a dedicated web-based application ‘EpiGEH’ which is described later in this article.

The plausibility of waterborne origin for each mAGE cluster is classified as: strong, probable, possible or indeterminate using another specific algorithm developed by SpFrance (Tables S2 and S3).

### Surveillance system management and implementation

**Figure 2:**
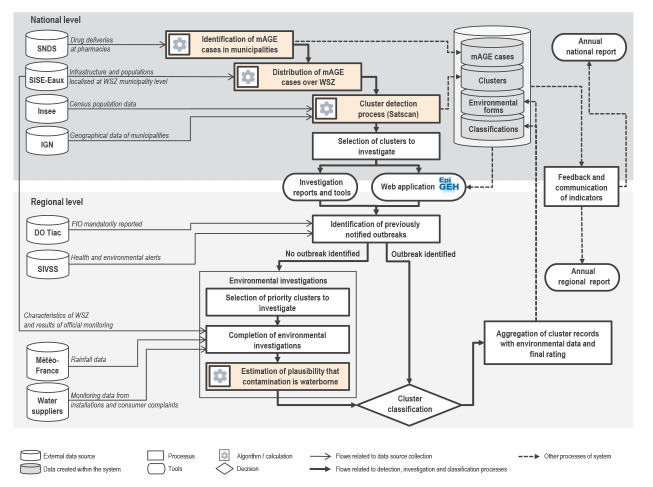
Main processes and flowchart, French national waterborne disease outbreak surveillance system

The French national WBDO surveillance system is run under the supervision of SpFrance (Figure 2). Its general framework and the requirements for the organisation of data collection between local stakeholders are detailed in a governmental directive [25]. The directive stipulates a 3-year start-up period (2019–2021) during which at least one cluster per year and per county must be investigated (i.e. almost 100 clusters per year nationally). The detection algorithm mentioned earlier is routinely updated every 4 months, over a retrospective period ranging from 3 to 7 months to take into account the consolidation delay for SNDS data (see Health data sources sub-section above).

DO Tiac: national food-borne infectious disease mandatory reporting system; FIO: food-borne infectious disease; IGN: French acronym for National Institute for Geographical and Forest Information; Insee: French acronym for National Institute of Statistics and Economic Studies; mAGE: medicalised acute gastro-intestinal enteritis; SISE-Eaux: French acronym for Health and Environment Water Information system; SIVSS: French acronym for Information System for Health Security; SNDS: French acronym for National Health Data System; WSZ: water supply zone.

During the surveillance system’s start-up period 2019 to 2021, the criteria to define priority clusters for environmental investigation were: (i) clusters associated with at least 10 excess mAGE cases and a risk ratio ≥ 3 and (ii) the repeated detection of clusters for the same WSZ.

In addition to routine detection, at the beginning of the start-up period, the detection algorithm was run over a 10-year period (2010–2019), and detected clusters were recorded in a national cluster database designed specifically for the new system, as historical cluster data are particularly useful to identify repeated clusters for a given WSZ and link them to previously notified WBDO.

### Data management and visualisation: EpiGEH web application

Standardised tools are used together with a web application (using the Shiny R package) named EpiGEH, developed by SpFrance to share analyses and investigation results between regional and national agencies [26,27].

**Figure 3:**
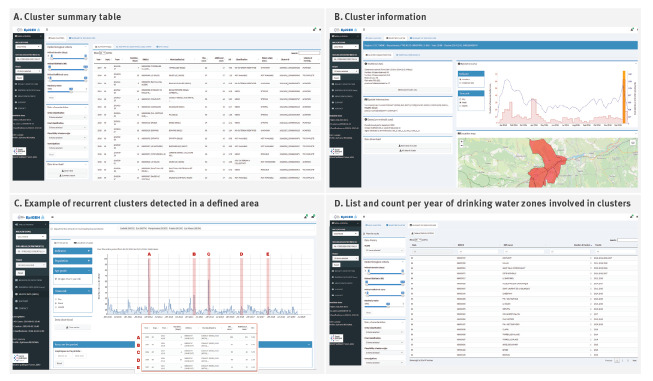
Overview of outputs available in EpiGEH

EpiGEH has two primary functions: (i) centralisation of data from WBDO surveillance system such as mAGE cases, environmental information forms, cluster epidemiological information and cluster classification and (ii) visualisation of detected clusters (Figure 3).

Data from environmental investigations and the classification of the level of plausibility of contamination from waterborne origin can be entered into the application using an interactive purpose-built form which is then integrated into the national cluster database.

The visualisation of detected clusters can be selected through EpiGEH’s summary table (Figure 3A) which contains the list of clusters, epidemiological criteria and main characteristics. Each cluster can also be visualised with an epicurve and by geolocalisation with associated WSZ (Figure 3B). Identification of repeat clusters and WSZ can be visualised either using a time series of mAGE cases or incidence (including by age group) in a specific geographic zone (Figure 3C), or consulting a list of WSZ and the number of associated clusters (Figure 3D).

### Ethical statement

We did not need specific ethical approval for the implementation of this surveillance system.

## Preliminary cluster detection results

The WBDO surveillance system was implemented in April 2019 and routine investigations are continuing as part of the initial 3-year start-up period. The preliminary results presented here regard historical clusters over 10 years (from January 2010 to December 2019).

**Figure 4:**
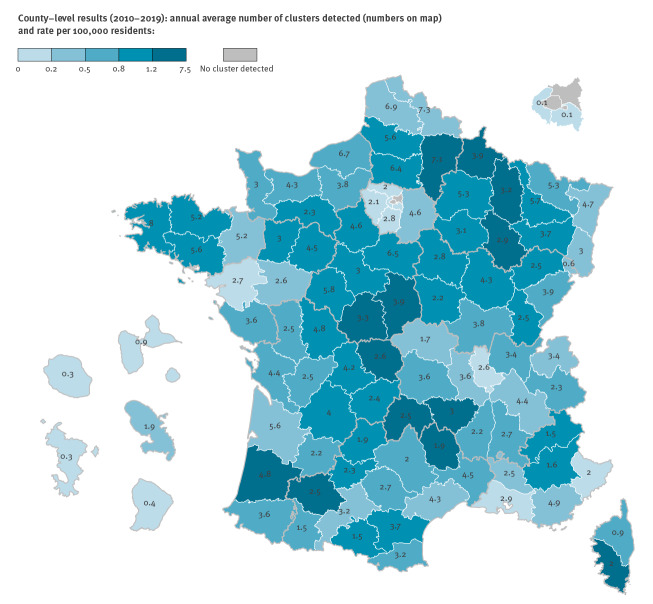
Number of priority clusters for environmental investigation (risk ratio ≥ 3, excess cases ≥ 10 and a minimum 3-day cluster duration), according to county, France, January 2010–December 2019 (n = 3,323)

Sources: GN−GEOFLA, 2016; Insee, 2014; Ministry of Health − SISE−Eaux, January 2020; National Health Insurance System database − SNDS, April 2020; Public Health France, April 2020.

Over the 10-year period, 9,193 clusters were detected, of which 3,323 (36.1%) were considered priority clusters for environmental investigation. The annual average number of priority clusters at the county level was 3.4 (range: 0.1–8.0) (Figure 4). The excess number of mAGE cases related to these clusters was estimated at 53,878 (82 excess cases per 100,000 residents over the 10-year period). The median proportion of mAGE cases involved in a cluster in the population served by the contaminated WSZ was 0.6% (25th percentile = 0.3%; 75th percentile = 1.3%).

**Figure 5:**
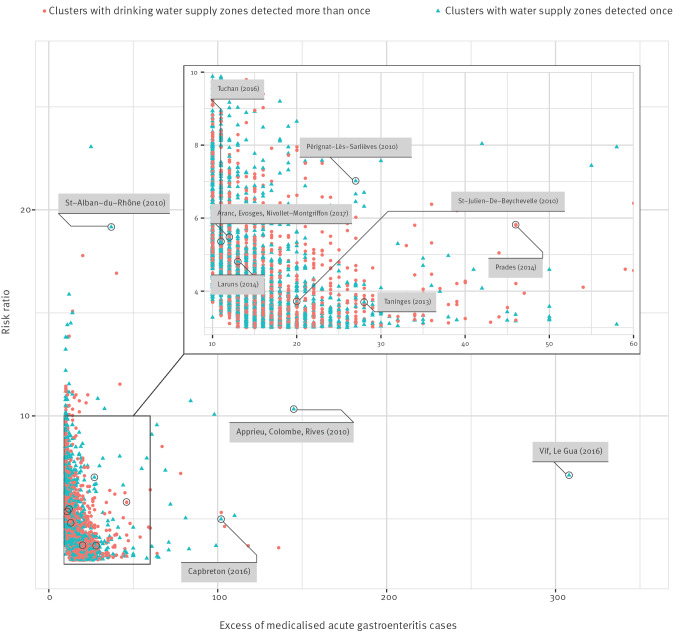
Priority clusters for environmental investigation (risk ratio ≥ 3, excess cases ≥ 10 and a minimum 3-day cluster duration) and a sample of notified acute gastroenteritis outbreaks, according to risk ratio and excess cases of medicalised acute gastroenteritis, France, January 2010–December 2019 (n = 3,323)

The median priority cluster duration stretched over 7 days (mean: 9 days). The majority of priority clusters had a risk ratio between 3 and 10, and a range of excess mAGE cases between 10 and 50 (Figure 5), although some clusters were characterised by a high risk ratio (i.e. > 10) and/or many excess mAGE cases (100–300) (Figure 5).

Between 2010 and 2019, 3,717 WSZ (15.4% of all French WSZ) were associated with priority clusters. Among these, 33.4% (n = 1,242 WSZ) were linked to repeat priority clusters.

Cross-referencing to detect priority clusters with previously notified WBDO between 2010 and 2019 showed that the majority (15/20) of these WBDO were detected by our new surveillance system (Table, Figure 5). Microbial contaminations of water resources, whether or not they were associated with treatment failure, were involved in half of these WBDO. Furthermore, for half of the WBDO, rainfall preceded the occurrence of cases. For the remaining detected WBDO, contamination of the drinking water network with wastewater or backflow from leaking sewage or from wastewater treatment plants was observed. The remaining five WBDO were not detected: two were associated with WSZ serving fewer than 200 inhabitants (Lachalade 2011 and Saint-Julien de Chapteuil 2014), one involved foreign tourists (notification occurred through the Early Warning and Response System (EWRS) from the European Centre for Disease Prevention and Control) (Bonifacio, 2017), one concerned a suspected chemical contamination with geosmine, associated with gastro-intestinal symptoms (Noyant, 2017), and the final one occurred in a military camp with no impact on the general population (cases not available in SNDS data) (Caylus 2017). All 15 clusters linked to WBDO were defined as having a ‘strong’ level of plausibility of waterborne origin when using the classification algorithm.

**Table:** Results from cross-referencing previously notified waterborne disease outbreaks with detected clusters, France, 2010–2019 (n = 20)

Notified waterborne disease outbreaks (municipalities and year)	Risk ratio	Observed cases	Excess cases	Duration (days)	Agents^a^	Circumstances/deficiencies
Apprieu, Colombe, Rives (2010)	10.3	160	145	12	Norovirus and rotavirus	Rainfall, microbial water-source contamination, chlorination treatment failure
Pérignat-Lès-Sarlièves (2010)	7.0	31	27	5	*Campylobacter*	Rainfall, chlorination treatment failure
St-Julien-De-Beychevelle (2010)	3.7	28	20	22	*Giardia duodenalis*, Adenovirus, *Cryptosporidium hominis, Escherichia coli, Staphylococcus aureus*	Suspected backflow from a WWTP
St-Alban-du-Rhône (2010)	19.2	39	37	7	Norovirus and Aichi virus	Backflow from a WWTP
Lachalade (2011)	ND	ND	ND	ND	Not available	Rainfall, chlorination treatment failure
Pleaux (2012)	2.3	40	23	25	Norovirus	Rainfall, treatment failure
Bourg Saint-Andéol (2012)	2.2	129	70	22	*Campylobacter*, adenovirus and rotavirus	-
Taninges (2013)	3.7	38	28	6	Adenovirus and rotavirus	Water-source contaminated with wastewater
Saint Julien Chapteuil (2014)	ND	ND	ND	ND	Negative results	Rainfall, no disinfection
Laruns (2014)	4.8	17	13	4	Norovirus, adenovirus, rotavirus sapovirus and Aichi virus	Suspected WWTP
Prades (2014)	5.8	55	46	4	Norovirus	Flooding
Pierrefort (2015)	14.0	8	7	3	Negative results	UV treatment failure
St-Firmin-en-Valgaudemar (2015)	4.3	12	9	18	Norovirus	Network contamination with wastewaters
Capbreton (2016)	5.0	128	102	8	Enterovirus and *Campylobacter*	Suspected backflow from a WWTP
Vif, Le Gua (2016)	7.1	358	308	10	Rotavirus and norovirus	Water contaminated with human faeces
Tuchan (2016)	5.4	14	11	7	Norovirus	Repairs to the network
Aranc, Evosges, Nivollet-Montgriffon (2017)	5.5	15	12	20	Rotavirus	Rainfall, microbial water-source contamination
Bonifacio (2017)	ND	ND	ND	ND	Not available	Backflow from a WWTP
Noyant et Lasse (2017)	ND	ND	ND	ND	Negative results	Drought
Caylus (2017)	ND	ND	ND	ND	*Cryptosporidium*	Water-source contaminated with animal dejection

ND: outbreak not detected by clustering method; UV: ultraviolet; WWTP: wastewater treatment plant.

^a^ In human stools.

## Discussion

For the first time in France, the implementation of a routine retrospective surveillance system for mAGE will make it possible to evaluate the level of plausibility of waterborne risk at the national, county, municipality and WSZ levels, including small WSZ serving between 200 and 500 inhabitants. Retrospective detection of clusters potentially associated with tap water exposure between 2010 and 2019 highlighted that, compared with previous voluntary notifications which identified a total of 20 WBDO over the same period, the new system identified at least 372 priority clusters per year, even if these two indicators are not fully comparable. This represents a notable improvement in the sensitivity of WBDO surveillance in France. Nevertheless, as environmental investigations of these clusters were limited to the 15 WBDO previously notified and detected by the algorithm, it was not possible to make an in-depth evaluation of the ‘plausible’ link with tap water contamination and to re-evaluate the real impact of WBDO to date in France. The lack of feedback to date concerning environmental investigations (partly because of the ongoing coronavirus disease pandemic) prevented us from being able to present data on these investigations here. However, we have planned a full report on the pilot phase (2019–2021) where these data will be presented.

This new innovative system relies on an integrated analysis of various big data sources and on environmental investigations. Moreover, it complements current official tap water monitoring and therefore contributes to ensuring improved tap water safety. Furthermore, it complements existing AGE surveillance systems not specific to waterborne diseases.

Although the new surveillance system underestimates the real health impact of WBDO - as only one third (33%) of AGE cases consult a doctor [28] and are subsequently identified as mAGE by the system - the identification of clusters represents an important new indicator which helps identify, through complementary environmental investigations, WSZ posing a recurrent microbiological risk for consumers.

Further assessment of priority clusters is a necessary step to complete this surveillance system. In the pilot study, the implementation time for environmental investigation was estimated at between 0.5 to 2 days per cluster [16]. When data finally become available, environmental investigations of priority clusters by various ARS and more detailed and frequent information by water suppliers should continue to provide important insights into the circumstances surrounding WSZ pollution, especially in terms of whether it is related to a resource, treatment and/or distribution incident. This information, even if only occasionally collected for a priority cluster, will help identify possible avenues for preventive actions.

Besides underestimating the real health impact of WBDO, the new system has other limitations. The first is its lack of reactivity, as the consolidation period for the health data (mAGE/SNDS) used is quite long (2 months). This delay between the onset of a WBDO and the ability to detect it, means that the new system is not suitable for promptly stopping the course of an outbreak, or indeed for preventing new cases. Instead, it is more adapted to identifying at-risk WSZ, preventing recurrence and contributing to prevention strategies. Maintaining existing surveillance through voluntary notification is therefore essential to ensure that public health authorities can respond quickly to WBDO.

Secondly, although the retrospective detection method used by the system is able to detect clusters potentially associated with a common route of exposure (i.e. tap water), any detection needs to be validated using additional environmental investigations focusing on, for example, microbiological data in tap water or potential technical incidents. Such investigations require coordination between the ARS and water suppliers and are time-consuming. Accordingly, new approaches to field investigations need to be developed. One example could be the use of daily rainfall data automatically collected by Météo-France, as rainfall is frequently and positively associated with infectious disease risk in tap water. These data could be used to evaluate the plausibility of waterborne contamination, as microbiological data in tap water are not immediately available for environmental investigations [29]. Indeed, heavy rainfall in the days before a detected cluster should be considered a consistent and reliable indicator for waterborne origin. Rainfall may also be one of the primary factors impacted in the context of climate change [8].

Given that the international classification for attributing waterborne origin to an outbreak [30] primarily uses microbiological and field-based epidemiological data, and that our system instead uses big data on health insurance complemented with data on environmental indicators (rainfall, laboratory data, historic information on recurrence, technical incidents, consumer complaints, microbiological pollution etc.), we needed to adapt the criteria for international classification to our system. To do this, we defined three critical classification criteria: (i) a vulnerability of the water production system to microbiological risk, (ii) a malfunction or malfunction-associated incident during the suspected exposure period and (iii) an external event aggravating a vulnerability or malfunction during the suspected exposure period (see Supplement). In our classification, priority clusters are classified as having possible waterborne origin (minimum level) if at least one of the criteria is met. The strength of association depends on meeting two or all three of the critical criteria. This classification will be re-evaluated when data from a sufficient number of environmental investigations become available.

Finally, beyond the objective of epidemiological surveillance, and despite the limitations mentioned above, the data on the 3,000 historical priority clusters identified by the new surveillance system can be used by health authorities - even in the absence of environmental investigations - to identify WSZ associated with repeated clusters over time, in order to implement or update water safety plans by water suppliers.

## Supplementary Data

493000 Supplement
