# Supplementary material for: Implementation of a national waterborne disease outbreak surveillance system: overview and preliminary results, France, 2010 to 2019
Source: Euro Surveill. 2021 Aug 26;26(34):2001466. doi: 10.2807/1560-7917.ES.2021.26.34.2001466 (PMC8393890; doi:10.2807/1560-7917.ES.2021.26.34.2001466)
Supplement: Supplement [file 20-01466_POUEY_Supplement.pdf]

# Supplementary materials

This supplementary material is hosted by *Eurosurveillance* as supporting information alongside the article "Implementation of national waterborne disease outbreak surveillance system in France: overview and preliminary results", on behalf of the authors, who remain responsible for the accuracy and appropriateness of the content. The same standards for ethics, copyright, attributions and permissions as for the article apply. Supplements are not edited by *Eurosurveillance* and the journal is not responsible for the maintenance of any links or email addresses provided therein.

**Table S1. Environmental investigation form**

Waterborne Disease Outbreak surveillance system

# ENVIRONMENTAL INVESTIGATION FORM

## FORM COMPLETION DATA

| | | |
|---|---|---|
| **Administrative entity:** | **ARS** | **External (water supplier, municipality)** |
| **Surname and first name:** | | |
| **Office :** | | |
| **Phone number :** | | |
| **E-mail :** | | |
| **Date of form completion or date of updating form :** | | |

## CLUSTER DATA

| | |
|---|---|
| **Region:** | |
| **County:** | |
| **Cluster start year:** | |
| **Study period:** | |
| **Cluster ID number:** | |
| **DWN(s) involved :** | |
| **Municipality(ies) involved :** | |
| **Cluster start:** | |
| **Cluster end:** | |
| **Beginning of suspected exposure period (31 days before start of cluster):** | |

## CLUSTER CLASSIFICATION

### ➡ Initial classification (known outbreak)

☐ NOT AVAILABLE ☐ WBDO ☐ FOODBORNE ILLNESS OUTBREAK ☐ AGE OUTBREAK in RETIREMENT HOME
☐ AGE OUTBREAK in COMMUNITY ☐ OTHER KIND OF AGE OUTBREAK ☐ NO OUTBREAK IDENTIFIED

### ➡ Plausibility of waterborne contamination

| | |
|---|---|
| 1 - Level of vulnerability calculated using algorithm: | *Under control / Potential / Strong* |
| 2 - Identified dysfunction calculated using algorithm: | *Not identified / Suspected / Occurred* |
| 3 - Existence of an aggravating event calculated using by algorithm: | *Yes / No* |
| **Plausibility calculated by the algorithm:** | **Strong / Probable / Possible / Indeterminate** |

**Plausibility retained:** ☐ STRONG ☐ PROBABLE ☐ POSSIBLE ☐ INDETERMINATE

### ➡ Final classification (after environmental investigations)

☐ NOT AVAILABLE ☐ WBDO ☐ FOODBORNE ILLNESS OUTBREAK ☐ AGE OUTBREAK in RETIREMENT HOME
☐ AGE OUTBREAK in COMMUNITY ☐ OTHER KIND OF AGE OUTBREAK ☐ NO OUTBREAK IDENTIFIED

*Comments on cluster:*

## 1 - VULNERABILITIES OF THE WATER PRODUCTION AND DISTRIBUTION SYSTEM regardless of the supposed exposure period

### PREVIOUS CLUSTERS DETECTED/OUTBREAKS

| Other detected clusters or known WBDO related to the DWN. | |
|---|---|
| - During the 3 years before the cluster | ☐ Yes ☐ No |
| - More than 3 years before the cluster | ☐ Yes ☐ No |

### IDENTIFIED VULNERABILITIES

| | |
|---|---|
| **Microbiological compliance rate over the last 3 years:** | ☐ Unknown<br>☐ <70%<br>☐ Between 71% and 95%<br>☐ 96% and over |

| Vulnerabilities identified: | |
|---|---|
| **At the water resource:** | |
| - Possible change in water quality as a result of heavy rainfall | ☐ Yes ☐ No |
| - Absence of protection perimeter | ☐ Yes ☐ No |
| - Other | ☐ Yes ☐ No |
| *If "Yes" above, specify the vulnerability here:* | |
| **At the water treatment plant:** | |
| - No disinfectant | ☐ Yes ☐ No |
| - Insufficient level of equipment (e.g., surface water resource with disinfection treatment only) | ☐ Yes ☐ No |
| - Insufficient safety equipment (e.g., absence of alarm or warning device at critical treatment points (filtration, disinfection, etc.) | ☐ Yes ☐ No |
| - Other | ☐ Yes ☐ No |
| *If "Yes" above, specify the vulnerability here:* | |
| **In the drinking water network:** | |
| - Areas without residual chlorine | ☐ Yes ☐ No |
| - Areas with long water residence times (> 48 hours) without rechlorination or dead zones | ☐ Yes ☐ No |
| - Network efficiency less than 70% | ☐ Yes ☐ No |
| - Poor protection/securing of water connections, especially fire hydrants (less than 70%) | ☐ Yes ☐ No |
| - Possibility of contamination at a reservoir | ☐ Yes ☐ No |
| - Previous back flow | ☐ Yes ☐ No |
| - Other | ☐ Yes ☐ No |
| *If "Yes" above, specify the vulnerability here:* | |

## 2 - MALFUNCTION OR MALFUNCTION-RELATED EVENTS during the suspected exposure period

| | | |
|---|---|---|
| **At least one consumer complaint characteristic of microbiological pollution** | ☐ Yes | ☐ No |

| | | |
|---|---|---|
| **Knowledge of microbiological non-conformities:** | | |
| - By the sanitary control authority | ☐ Yes | ☐ No |
| - By the operators in charge of the water treatment or production systems | ☐ Yes | ☐ No |
| - Samples with more than 10 CFU of Enterococci (EN) + Escherichia coli (EC) / 100ml for the sum of the samples on a given day | ☐ Yes | ☐ No |

| | | |
|---|---|---|
| **Knowledge of a malfunction at the resource, treatment plant or distribution network:** | | |
| - Disinfection failure at treatment plant | ☐ Yes | ☐ No |
| - Rechlorination failure at reservoir and distribution network | ☐ Yes | ☐ No |
| - Event at treatment plant (to be specified below) | ☐ Yes | ☐ No |
| *If "Yes" above, specify which event here:* | | |
| - Event at distribution network (to be specified below) | ☐ Yes | ☐ No |
| *If "Yes" above, specify which event here:* | | |
| - Event at reservoir (to be specified below) | ☐ Yes | ☐ No |
| *If "Yes" above, specify which event here:* | | |
| **-> Malfunction considered serious enough to be associated with the outbreak** | ☐ Yes | ☐ No |

| | | |
|---|---|---|
| **Knowledge of abnormal variation of a parameter measured continuously or frequently:** | | |
| **<u>At the resource:</u>** | | |
| - Turbidity | ☐ Yes | ☐ No |
| - pH | ☐ Yes | ☐ No |
| - Other | ☐ Yes | ☐ No |
| *If "Yes" above, specify which parameter here:* | | |
| **<u>At the treatment plant or at the exit of the plant:</u>** | | |
| - Chlorine demand | ☐ Yes | ☐ No |
| - Turbidity | ☐ Yes | ☐ No |
| - pH | ☐ Yes | ☐ No |
| - Other | ☐ Yes | ☐ No |
| *If "Yes" above, specify which parameter here:* | | |
| **<u>At distribution unit level (network, reservoirs, rechlorination station):</u>** | | |
| - Chlorine demand | ☐ Yes | ☐ No |
| - Turbidity | ☐ Yes | ☐ No |
| - pH | ☐ Yes | ☐ No |
| - Other | ☐ Yes | ☐ No |
| *If "Yes" above, specify which parameter here:* | | |
| **-> Variation considered important enough to be associated with the outbreak** | ☐ Yes | ☐ No |

## 3 - EXTERNAL EVENTS AGGRAVATING VULNERABILITY OR MALFUNCTION during the suspected period of exposure

| | |
|---|---|
| **Weather Trend: Heavy rain** | ☐ **Yes** ☐ **No** |

| | |
|---|---|
| **Agricultural situation: period of manuring and manure application** | ☐ **Yes** ☐ **No** |

| | |
|---|---|
| **Unusual event at water intake or catchment basin:** | |
| - Presence of dead fish (surface water) | ☐ **Yes** ☐ **No** |
| - Increased pollution at the catchment area (overflowing sewage treatment plant, presence of contaminated water mass, etc.) | ☐ **Yes** ☐ **No** |
| - Other (accidental spill in the catchment supply pond, report of animals refusing to drink, repair or maintenance operations, break-in at the immediate protection perimeter, etc.) | ☐ **Yes** ☐ **No** |
| *If "Yes" above, specify the unusual event here:* | |

| | |
|---|---|
| **Unusual event in drinking water network:** | |
| - Return of contaminated water (unlawful connection to the internal network of a wastewater treatment plant or to factories with an internal technical water network) | ☐ **Yes** ☐ **No** |
| - Other event at the water network (e.g,. fire hose racks being unlawfully removed, fire hydrants being used for fires or firefighting training, etc.) | ☐ **Yes** ☐ **No** |
| *If "Yes" above, specify the unusual event here:* | |
| - Other event near the water network (e.g., work on other underground networks: electricity, gas, sewerage, etc.) | ☐ **Yes** ☐ **No** |
| *If "Yes" above, specify the unusual event here:* | |

| | |
|---|---|
| **-> External event believed to be associated with the outbreak (if Yes: waterborne plausibility is classified as possible, even in the absence of identified vulnerability or malfunction)** | ☐ **Yes** ☐ **No** |

**Table S2. Level of plausibility of waterborne infection/contamination according to the level of vulnerability of the water production and distribution system to microbiological risk, to malfunction or to malfunction-related events.**

| Vulnerability of the water production and distribution system to microbiological risk * | Malfunction or Malfunction-related event * | Waterborne plausibility |
|---|---|---|
| Strong | Occurred | Strong |
| Potential | Occurred | Strong |
| Under control | Occurred | Strong |
| Strong | Suspected | Strong |
| Potential | Suspected | Probable |
| Under control | Suspected | Possible |
| Strong | Not identified | Probable/Strong * |
| Potential | Not identified | Possible |
| Under control | Not identified | Indeterminate/Possible * |
| Irrespective of the level of vulnerability or the occurrence of a malfunction, knowledge of an event characteristic of pollution ** | | Strong |

* Special situations where an external event is directly related to a known vulnerability of the water production and distribution system: (i) resource sensitive to heavy rainfall and heavy rain, (ii) resource with no protection perimeter and spreading of liquid manure, (iii) WDZ with no disinfectant and increase in pollution, (iv) an area in the WDZ with no chlorine and usual event in the network in these areas (e.g., repairs, maintenance).

** Existence of a contaminated network return water or presence of dead fish at the water intake.

**Table S3. Level of vulnerability and malfunctions depending on an external event**

| | External event aggravating vulnerability or malfunction during suspected exposure period ** | |
|---|---|---|
| | **No event observed** | **Event observed** |
| **Vulnerability of the water production and distribution system to microbiological risk** | Strong | Strong |
| | Potential | Strong |
| | Under control | Under control |
| **Malfunction or malfunction-related event** | Occurred | Occurred |
| | Suspected | Occurred |
| | Not identified | Not identified |
